# Unpacking rights in indigenous African societies: indigenous culture and the question of sexual and reproductive rights in Africa

**DOI:** 10.1186/1472-698X-11-S3-S2

**Published:** 2011-12-16

**Authors:** Chi-Chi Undie, Chimaraoke O Izugbara

**Affiliations:** 1 Population Council, General Accident House, Ralph Bunche Road, P.O. Box 17643, 00500 Nairobi, Kenya; 2 African Population and Health Research Center, APHRC Campus, Kirawa Road, off Peponi Road, P.O. Box 10787, 00100 Nairobi, Kenya

## Abstract

**Background:**

Modern declarations on human rights have often proceeded without reference to the cultural content of rights, the existence of rights in African indigenous backgrounds, and the embodiment of certain key rights in the community itself. This paper is an attempt at developing an ‘inventory’ of rights in African cultures as a prelude to the generation both of a holistic theory of rights as well as a research agenda that can recognize the multifaceted nature of rights.

**Methods:**

We use an interpretive ethnographic approach built on three sources of data: 1) our continuing ethnographic work among two distinct ethnic groups in southeastern Nigeria – the Ubang and the Igbo; 2) informal conversational interviews with individuals from a range of African countries; and 3) a review of relevant literature based on African cultures which provides a context for some of the issues we raise.

**Results:**

An examination of selected indigenous rights, entitlements, or privileges among the Ubang and Igbo illustrates indigenous culture as a key, but often neglected, axis of rights, as a critical framework for understanding human relationships with rights, and as a resource for, and challenge to, contemporary programmatic efforts focusing on universalized notions of rights. Understanding or interpreting rights in African settings within the framework defined by contemporary human rights discourse poses steep challenges to making progress in the realization of sexual and reproductive rights.

**Conclusions:**

Despite the potential dangers of privileging group rights over individual rights, when important rights are vested in the community; rights, entitlements, and privileges can also be recognized through community experiences, and realized through engagement with communities. Building on communal conceptualizations of rights in order to realize an even wider range of rights remains a largely unexplored strategy which holds promise for the achievement of sexual and reproductive health rights.

## Background

The concept of ‘rights’ has become an increasingly contentious term in African settings, as elsewhere. The individualized manner in which universal rights are often framed explains much of the controversy in African contexts, which many would describe as more ‘community-oriented’ than ‘individually-oriented’. This taken-for-granted categorization of African and other milieux has however begun to be problematized in the literature, and its dangers as a less than holistic perspective are now obvious. For instance, Corrêa, Petchesky and Parker [1] draw our attention to the fact that rights are neither completely communal, nor totally individual. Rejecting the dichotomy between the individual and the community, they instead put forth a different vision – one that “encompasses *both* singularity and interdependence … [E]conomic and social rights accruing to communities (for safe water, health care, livelihoods) are ultimately about the individual bodies that need these resources to live. Rights are always individual and social at the same time, just as persons are” ([1]. p222).

This refreshing observation is also at the heart of our arguments elsewhere [2], that individual rights in many African contexts are not stand-alone properties or events. They are linked to, and embodied in the community, which often sees the individual as part of it. The African Charter on Human and Peoples’ Rights [3] also recognizes the interrelationship between the community and the rights of individuals and peoples. It thus clearly states that the rights and freedoms of individuals are only meaningful when exercised with due regard to the security, values, and interests of the community. Although rights are often simultaneously individual and social, the dissimilar weights assigned to each of these in varying contexts highlight the need for more culturally-sensitive approaches to the realization of rights in different societies.

In this paper, we continue from our previous discussion [2] on how rights are ‘weighted’ and constituted in indigenous African cultures. We aim to deepen the understanding of ‘rights’ as a socio-cultural axis upon which many discourses turn. Such an understanding has become particularly critical given unsituated claims that “no individual, family, kinship group, clan or community ‘owns’ the body of another person” ([4]. p115). This paper is thus an attempt at developing an ‘inventory’ of rights in African cultures as a prelude to the generation both of a holistic theory of rights as well as a research agenda that can recognize the multifaceted nature of rights.

The myriad contestations around rights have often invisibilized the existence of rights or entitlements within indigenous cultures. Consequently, modern declarations on human rights have often proceeded without reference to the cultural content of rights, the existence of rights in African indigenous backgrounds, and the embodiment of certain key rights in the community itself. Our interest thus centers on ‘indigenous’ rights for, as we have previously contended [2], the strong allegiance of many to their indigenous cultures and the generally weak and unrealistic modern frameworks for the realization of rights often lead to their search for rights within the indigenous cultural space. The supposedly protective formal law courts, the idealized shielding and fair modern state, and the global human rights movements often have limited relevance for the everyday realities of people in local communities in Africa. Recourse to indigenous axes of rights has thus remained the major tactic through which individuals stake, guarantee, and achieve claims to their rights. An examination of how rights are framed from the perspectives of local communities, it is hoped, will provide deeper understanding of indigenous world-views on sexual and reproductive health rights, and therefore, of how to take steps toward their realization.

In focusing on ‘indigenous’ rights, we recognize the complexity of the notion of ‘tradition’, given the vast structural changes that African cultures have experienced between the pre-colonial era and the present time. Borrowing from Gyekye [5], Nzegwu [6] notes the politics of customs (upon which ‘indigenous’ phenomena are based), contextualizing this politics while observing that “there is nothing static or stable about traditions and customs”, rather, “the root of the problem lies in the way that present generations have chosen to preserve their customs or traditions” ([6]. p139). In Gyekye’s words: ‘the preservation of [a belief or practice], in part or in whole, would depend very much on the attitude the new generation adopts toward it. … This means that continuity and survival of a pristine cultural product depends on the normative considerations that will be brought to bear on it by subsequent generations’ ([5]. p221). As Nzegwu emphasizes, ‘Gyekye’s point is that customs are rarely received and preserved in their old forms. This tells us that our focus should be on the attitude and normative values that [individuals bring] to bear on receiving and deploying these customs, rather than on the customs themselves’ ([6]. p139).

In our discussion, we will devote our attention to the Ubang and Igbo customs themselves (as many readers will presumably be unfamiliar with them) and the attitudes and normative values surrounding them.

## Methods

We employ an interpretive ethnographic approach to ‘unpacking’ rights in the paper – meaning that “we are *interpreting* what our ethnographic study has shown us is there, in the situation where the action is taking place. … Interpretive ethnography requires inference, even speculation, but these inferences and speculations must be grounded in observation and inquiry, in depth, in situations … where we have become familiar figures and can be treated casually by our informants” ([7]. p. xii). We build this approach on three sources of data: 1) our continuing ethnographic work among two distinct ethnic groups in southeastern Nigeria – the Ubang and the Igbo; 2) informal conversational interviews with individuals from a range of African countries (the informal conversational interview – also referred to as ‘ethnographic interviewing’ – “relies entirely on the spontaneous generation of questions in the natural flow of an interaction, often as part of ongoing participant observation fieldwork” ([8]. p342)); and 3) a review of relevant literature based on African cultures which is useful in explaining some of the issues we raise.

The first author initially carried out fieldwork among the Ubang people of Obudu in 2001, and has continued to conduct participant observation in the Obudu context since then, being married and having had children within this setting. We are both members of the Igbo community, which happens to be the ethnographic field site of the second author, who has conducted fieldwork among the Ngwa Igbo in particular since 1996. The Ubang and Igbo homelands are both in southeast Nigeria. Over the years, our research has involved an assortment of qualitative techniques, namely ethnographic observation and informal interviews. We have spent long periods of time living with and among the Ubang and Ngwa Igbo peoples, respectively, participating in their lives and social activities, including marriage ceremonies, funerals, community development activities, and holding individual and collective discussions with men and women in the communities. Taken together, our studies, though begun at different times and located in different cultural settings, address cultural constructions of, and the relationships between, gender, sexuality, the body, and rights in local communities. Although we have invariably worked independently, we have soon discovered that we were asking similar questions: How are rights constituted indigenously, and how important are local constructions of sexuality, gender, the body, and belonging in informing the realization of rights and entitlements in local Nigerian cultures? In this paper, we draw on the data which we have collected from these two cultural contexts to discuss the framing of rights and freedoms, and the implications of these constructions for pursuing the current sexual and reproductive health rights agenda.

In the course of our work, we continue to have the privilege of interacting with Africans from all over the continent, either professionally or personally. Our interest in sexual and reproductive health often tends to steer our conversations with those we meet toward various aspects of this subject. In the process, we have garnered an ‘inventory’ of sorts – examples and/or case studies of sexual and reproductive health rights as they manifest themselves in the present day. In comparing these examples to older and current ethnographic literature, we are repeatedly fascinated to observe how little has changed (over the last half a century or so) in regard to attitudes and normative values despite the massive transformations in African settings brought about by modernity.

We begin by presenting examples of indigenous rights which remain relevant in contemporary times to gain a sense of how rights in general are conceptualized in the two contexts in question. We later examine instances of how these conceptualizations inform concepts of sexual and reproductive health rights today. In approaching the article in this fashion, our aim is two-fold: 1) to draw attention to existing indigenous rights in order to stimulate dialogue and thinking around how synergies may be found (or not) between them and sexual and reproductive rights, thereby advancing this rights agenda in the region; and 2) to examine attitudes and normative values that many individuals bring to bear in deploying their sexual and reproductive rights (whether indigenous or not).

## Results and discussion

### Rights in Igbo and Ubang worldviews

Neither the Ubang nor the Igbo have a direct term for ‘rights’. Rather, they describe rights using phrases that nonetheless reflect the centrality of rights in the lives of individuals and communities. Among the Igbo, rights are described in terms of people’s inalienable due; *ihe duru ya*: one’s rightful entitlements; *oke duru ya*: one’s rightful share. A key manner in which the Ubang refer to ‘rights’ involves the notion of privileges – *libuo li ye*, which is translated to mean ‘my privilege’. In the two cultures, entitlements to certain rights and freedoms are shaped by a variety of factors. According to the Igbo, being born a human and, particularly, an Igbo, are among the key factors that entitle one to certain rights. The Igbo belief in the centrality and inalienability of certain rights to human nature is exemplified in expressions such as: *Madu bu Madu*, *ma ha cha ucha ma ha jinji* (‘humans are humans notwithstanding their skin color’). While the Igbo and the Ubang alike are wont to conceive of the rights and freedoms of individuals in universalistic and egalitarian ways, they at the same time espouse the notion that certain characteristics (including gender, generation, lineage membership, birth order, experience, etc.) place some in good stead to enjoy certain unique rights and freedoms. These freedoms and rights, however, also come with responsibility. The indigenous notions of rights among the Igbo and Ubang derive in large part from what Nickel [9] describes as collective norms related to lived and actual human virtues, and acceptable ethical norms or natural rights grounded in convincing reasons and sensibilities. Because they tend to proceed from lived experiences and historical realities, indigenous notions of rights are hardly abstract. Yet, modern human rights declarations that proceed from theoretical ideas about human beings, who are born free and equal in dignity and rights and endowed with reason and conscience (see, for example, the Universal Declaration of Human Rights) [10], do not, in reality, contradict local Ubang and Igbo notions of rights. What they often lack is grounding in the lived and dynamic, everyday social experiences of the people. In many African contexts, indigenous cultural values continue to present peoples and groups, rather than individuals, as the framework for engaging and relating with questions of rights and entitlements. Indeed, in interviews with several respondents who were asked for Ubang, Obudu expressions to convey the term ‘rights’, each interviewee highlighted the severe challenge of translating a term (or phrase), which in English had a strongly individualistic connotation (from their individual perspectives), while Obudu understandings of the term were said to be much more communal. While the dynamism of African cultures is not contested, forces continuously work to promote conservatism. Stamp [11] notes that despite laudable strides in formal education and modernization, issues of rights and privileges for men, women, and children are still largely constructed in indigenous terms in most parts of contemporary Africa. Importantly, however, cultures are also often open to change, indicating their inherent capacity to accommodate and anticipate newer and abstract notions of human progress, community life, and governance.

### Indigenous rights: some examples

Our discussion of selected indigenous rights, entitlements, or privileges among the Ubang and Igbo is centered on five themes, namely: socio-economic, political, personal security, reproductive, and sexual rights. These are not meant to be exhaustive (rather, they are indicative of the range of rights which the cultures in question potentially offer, and of potential openings to the achievement of other rights). Nor are they meant to highlight local norms of rights and entitlements as a panacea for all the issues associated with realizing rights, or for the myriad predicaments facing modern states. Our point here is to illustrate indigenous culture as a key, but often neglected, axis of rights, as a critical framework for understanding human relationships with rights, and as a resource for, and challenge to, contemporary programmatic efforts focusing on universalized notions of rights. Furthermore, our point is to demonstrate the steep challenges posed by understanding or interpreting rights in African settings within the framework defined by contemporary human rights discourse. Nzegwu describes this framework as one that “pits the rights of individuals against the norms of cultures and traditions”, observing that “tensions have steadily arisen between those eager to secure the individual rights of the people over the customs and values of cultures that violate those rights” ([6]. p1). Our premise is that, in indigenous contexts, many individual rights are virtually inseparable from the communities which, through customs, confer the rights in the first place. This reality is a recurrent theme that runs through each of the examples of rights provided in this section.

Lastly, we are also aware that our data are drawn from only two cultures and that other interpretations of the materials highlighted here are possible:

#### i. Socio-economic rights

Given the importance of agriculture among the Igbo and Ubang, land ownership is central to any exploration of socio-economic rights among them [12]. Relationships with land in the two cultures are based on three principles: 1) that land ultimately belongs to the community and cannot be alienated from it without its consent; 2) that within the community, the individual has security of tenure for the land required for his/her compound, gardens and farms; and 3) that no member of the community shall be without land [13]. Essentially, security of tenure is available to all individuals for land required for residential and farming purposes. This right to land, however, must be obtained from a lineage – usually the lineage of one’s father (for men) or of one’s husband (for women). In other words, an individual is not entitled to land as a consequence of his/her desire to be a land owner or to have access to land – but an individual is automatically entitled to land because of his/her status as a lineage member.

A person’s affiliation with his/her mother also guarantees certain socio-economic entitlements and privileges. Indeed, when in one’s maternal lineage, one is referred to by a distinct name – *okene* for the Igbo, and *nwang oshie* for the Ubang. The terms can be roughly translated to mean ‘child of the mother’s natal village’. In her ethnography of the Ubang, Undie [14] explains that within the patrilineage of his/her mother, a person is indulged and granted a level of leeway that is unparalleled elsewhere. This indulgence of the ‘child of the mother’s natal village’ among the Ubang is demonstrated by the extravagant manner in which a *nwang oshie* plucks all kinds of fruit from the family economic trees when s/he visits the mother’s patrilineage. S/he seizes and claims any chickens seen roaming around to everyone’s delight. While such behavior would be regarded as excessive in one’s patrilineage, and would be met by reprimand, it is praised, applauded, and meets with genuine pleasure and amusement in the natal village of one’s mother. A *nwang oshie* can expect financial and other support from his mother’s natal village during austere times, or during marriages and funerals. Among the Ngwa-Igbo, the *okene* is the equivalent of the *nwang oshie*. The *okene* is ascribed with certain mystical powers and seen as potentially capable of bringing both curses and blessings to his/her mother’s natal village. The Ngwa thus say that ‘*Onu okene bu onu mmuo*’ (‘An *okene's* word is the word of the gods’). To harm an *okene*, refuse his or her legitimate request, or to fail to support him or her in a time of need is considered evil. It is also an abomination for the blood of an *okene* to be shed in the vicinity of, or presence of persons from his/her mother’s natal village. The *okene* is thus not only entitled to absolute protection by members of the mother’s natal community, but also to a life of indulgence while in their midst. Uchendu [15] describes the position of the *okene* as follows:

The sister’s son enjoys a special status in the lineage of the mother’s brother. He may not spill his blood while he is there; he may not be so provoked that he decides to bounce his buttocks on the ground, nor may he stamp a pestle on the ground. For him to do any of these is taboo, an offense to the earth-goddess. It is his [mother’s lineage] who must bear the consequences of his action. While he stays with his mother’s brother, he is treated with much affection and allowed a wide latitude in his behavior. His playmates are warned against the danger of making him spill his blood ([15]. p67).

The wide latitude in behavior granted to an *okene* is evidenced by the privileges he has to claim any item from his maternal uncle’s home without asking for permission, for example – or his ‘poaching’ of palm wine (an act that is met with exhilaration in this context) from his maternal uncle’s lineage when he runs short of this commodity for entertaining his guests.

Despite the nearly fifty-year span between *okene* as observed by Uchendu and *okene* in contemporary times, this description continues to hold true today. The authors of the current paper being *umu okene* (plural) in the natal homes of their mothers are constantly reminded of their rights to protection and excess in these communities. One is often told to take, eat, and drink whatever one wants; say whatever one feels like saying; and make any kind of demands on members of the mothers’ lineage. We have both also experienced situations where our *okene* status permitted us to infringe key social norms. Furthermore, our children enjoy the same privileges in their own mother’s patrilineages. Continuity of the *okene/nwang oshie* institution is also observed in Ubang, where the first author has repeatedly observed individuals of this status visiting their mothers’ natal villages and engaging with their relatives in this way, since 2001.

#### ii. Rights to political participation

The patrilineage provides most rights that the Ubang and Igbo have in regard to indigenous politics and political positions. While it is difficult to speak of a common political organization system among the Igbo, a basic feature of the various political systems that existed among different traditional Igbo groups was, and continues to be, the powerful role of patrilineages or matrilineages. Traditional Igbo political life is organized around membership in, and affiliation with, kinship group associations, such as age-grades, men's associations, women's associations, and titled persons’ associations. Generally, ascension to any important position in traditional Igbo society requires approval from kinship, age-grades, and other associations of which one was a member. Access to political privileges also depends on membership in clearly identified key groups. The diffusion of political authority through key kinship groups, age sets, title societies, sex-based associations, and professional groups ensures that members of the society have different points of access to political decision-making. It is thus common, even in contemporary Igbo society, for one’s kinship groups, age sets, chieftaincy title, professional, sex-based, and other groups to fight for redress on behalf of their wronged members.

The Ubang provide another good example of the critical importance of membership in, and affiliation with, kinship groups for political participation and access to power. In her natal home, regardless of marital status, an Ubang woman is referred to as *nwang abeh* (meaning ‘umarried [female] child’ or ‘spinster’). Within her marital home, however, a woman is referred to as *unyelube* (‘married woman’). This distinction is important as the rights of a woman within her natal home vary remarkably from her rights within her marital home.

The Ubang explain that the *bebuang abeh* (plural of *nwang abeh*) run their own governing council, which is separate from that of Ubang men. This governing council focuses on the issues of lineage women in general, but is also very influential in wider community matters. When necessary, *bebuang abeh* are called back from their marital villages in order to participate in the important political activities of their governing council. The rights associated with this political group include the resolution of major and delicate family and inter-community conflicts (such as land disputes, manslaughter, etc.) that men of the patrilineage have been unable to resolve. Although this is a rare occurence, the ‘children of the mother’s natal village’ described in the previous sub-section, may also be called upon to serve as arbitrators during particularly difficult cases in the community. Given their subordinate position vis-à-vis *bebuang abeh* (village daughters), the political rights of village wives are comparatively negligible, although wives whose status has been elevated over time (as a result of their economic prowess, or the achievements of their children, for instance) play key roles in the smaller family setting, and wives as a collective group can sometimes exert political pressure within their marital villages.

Rights to political offices and power among Ubang men, on the other hand, are dependent upon family membership. The political spheres in which men may engage and the levels that they may attain within these spheres are pre-determined along familial lines. Every male Ubang indigene has access to some form of political power based on the family into which he was born. One might be born into the group of families pre-designated as ‘king makers’, or as village heads, or as heads of a particular secret society, for instance. A village head, though charged with the responsibility of overseeing the community, may nonetheless be subordinate to the head of a secret society when operating within the area of spiritual matters and vice-versa. In this way, ingenious checks and balances are built into the Ubang political structure to ensure power-sharing amongst the various indigenous, male-dominated political groups.

#### iii. Rights to personal security

While the patrilineage is a key channel through which rights flow to an individual, it is also paradoxically known as a relatively hostile environment where its sons and daughters are concerned in both Ubang and Igboland. Brotherliness and rivalry co-exist in the context of one’s patrilineage. The maternal home, conversely, is a ‘safe house’ of sorts – a place of safety and security. It is traditionally understood that while one has an unequivocal right to total protection in the maternal home, in the paternal home, one is more vulnerable to various kinds of attacks, as the consequences of such attacks are less stringent. While untimely death is a serious issue whether it occurs in the maternal or paternal home, the murder of a ‘child of the mother’s natal home’ is considered more grievous than that of an individual within his/her paternal home. In the maternal home, repercussions believed to affect the entire community are incurred when a ‘child of the mother’s natal home’ is gravely offended or murdered, and special sacrifices must be offered thereafter. The Ubang, for instance, say that “One can never kill a *nwang oshie*”, demonstrating the personal security that the mother’s patrilineage provides. One flees from one’s own patrilineage, however, after having committed murder there (whether intentionally or not), and when faced with accusations of sorcery. The place of refuge in such situations is the mother’s patrilineage, which provides full support for the accused until the sentence imposed elapses.

Izugbara [16] observes that the primary place of refuge for young schoolgirls who experience out of wedlock pregnancy among the Ngwa-Igbo is often the villages of their mothers. Ordinarily, such girls would be the targets of mistreatment, and sometimes violence in the hands of their parents and immediate family members. But fleeing to their mothers’ villages where they are above blame, condemnation, and rebuke shields them from any form of persecution, harm, and abuse. Achebe’s *Things Fall Apart *[17], set in traditional Igbo society, also drives home the critical role played by lineage groups in protecting the individual. Okonkwo, the novel’s protagonist, is exiled for seven years to Mbanta, the village of his mother, after his gun accidentally discharges a bullet which kills a sixteen year-old child. In appreciation of the protection and refuge which his mother’s village offered him during this period of crisis and insecurity, Okonkwo named the first daughter born to him in exile ‘Nneka’, which means ‘Mother is supreme’.

#### iv. Sexual and reproductive rights

The indigenous sex institutions of the Igbo and Ubang underscore the ability of both cultures to make concessions in regard to sexual and reproductive issues. They also emphasize the important role assumed by the community in conferring rights even in regard to sexual matters. In his description of sex institutions in Igboland, Uchendu [18] observes that “There is no emphasis among the Igbo on sexual services being *exclusive* and confined to husband and wife. All that the culture demands is that sex be institutionalized” ([18]. p193). This assertion can be made for the Ubang as well, with their socially-sanctioned *utin* institution in which women and men may opt to maintain lovers with the knowledge of their community [14]. Although in theory, a (male) husband has the right to sexual exclusivity where his wives are concerned, the institution of concubinage enables women and men to circumvent this right, exerting their rights to sexual partners outside of marriage. A key characteristic of this institution is its openness. While covert extra-marital sexual relations are regarded as marital infidelity, this is not the case in regard to concubinage, which involves some simple rites that grant it societal approval.

Children borne out of a concubinage union in these contexts are automatically affiliated with the husband of the mother of the child, rather than to the biological father who, in not being the one to have paid bridewealth, gives up the right of filiation. As in the case of many African cultures, the payment of bridewealth legalizes marriage among the Ubang and Igbo, and reassigns certain rights in a woman from her patrilineage to that of her husband. Of note is the reassignment of the right to a woman’s potential fertility, which Uchendu [18] describes as “patrilineally centered and … therefore indivisible” ([18]. p189). As he explains, “Who ‘fathers’ the child is irrelevant. What is crucial is ‘who paid the bridewealth’. Since the right to a woman’s fertility is acquired at marriage, any children she may bear are filiated to the man or woman who acquires the right: it makes no difference whether this individual begets these children or not” ([18]. p189). This point is important as it departs from contemporary ideas of reproductive rights as having solely involving individual or couple decisions about the number of children to have, for instance, overlooking the role that communities may play in ascribing child affiliation.

Furthermore, in Igboland, bridewealth payers may be female or male. Women that can raise the bridewealth payment also have the right to marry wives, with the attendant rights to the bride’s fertility that marriage entails [15,18]. Children born within such marriages (via sexual relations between the bride and her chosen paramours) are automatically affiliated with the bride’s female husband, rather than to the children’s biological fathers. Although woman marriage is not an indigenous institution of the Ubang, the ‘social’ fatherhood that Uchendu describes applies to this culture as well: should a biological father fail to pay the bridewealth of his child’s mother, the child in question is affiliated with its maternal grandfather. The social logic here is, again, that bridewealth legalizes marriage and resolves the issue of children’s filiation. Without the bridewealth payment, the right to a woman’s potential fertility is not transferred to the lineage of her paramour(s). Children borne out of such unions therefore belong to the father of the woman concerned, rather than to their actual biological father.

### Indigenous notions and the realization of contemporary rights: some examples

The more traditional/indigenous conceptualizations of rights described above are no doubt challenged by vast changes occurring as a consequence of education, migration, urbanization, and the spread of markets, to mention a few factors. It is therefore all the more curious to us that indigenous ways of thinking about rights hold sway at all in African settings. And yet they do. Our analysis of the data we have collected and pulled together from different sources shows that the embeddedness of individuals in their communities continues to affect the choices they eventually make despite their knowledge of the range of choices/rights that are theoretically available to them. This, in turn, affects the realization of rights as outlined within international conventions. We now explore several examples that demonstrate ways in which reproductive rights in particular are playing out in African settings in contemporary times:

#### i. What’s in a name?

Assisted reproductive technologies are playing an increasingly important role in expanding reproductive choices in sub-Saharan Africa. Indeed, given the critical importance attached to parenthood in African societies, some may find it surprising that the use of these technologies is not more deeply entrenched. A series of informal conversational interviews on the specific issue of artificial insemination (aided by anonymous sperm donors) furthers our understanding of conceptualizations of reproductive rights and how these affect the realization of individualized concepts of rights. Through these conversations, we were educated on the intricacies of naming in Ugandan cultures, for example. In the course of getting acquainted, Ugandans are said to ask about each other’s *birth places*, rather than for each other’s names. The question ‘*Where were you born?*’ is not to be taken literally, however, as its actual translation is more along the lines of ‘*What is your clan name?*’ – one’s clan name being inextricably linked to one’s clan or natal home. Clan names are chosen by one’s maternal and paternal grandparents, who officially send in the chosen names via a brown envelope. One informant recounted an instance of an infant that remained without a name for a considerable amount of time as one grandparent was out of the country and had to wait until she found someone returning to Uganda through whom she sent the clan name in the conventional brown envelope. The name given by grandparents solidifies one’s position within the clan and makes apparent one’s entitlements (e.g., to land, to a befitting marriage or burial, etc.) within the clan context.

Naming is thus a major source of concern for Ugandan women considering artificial insemination with an unknown sperm donor. An anonymous donor raises a series of issues concerning the unborn child. How will s/he be named, and by whom? What entitlements or privileges will s/he have, given that the father (and therefore, the father’s patrilineage) is unknown? The concern that the rights of such a child would be diminished gives some women pause when considering exercising their own reproductive rights through this means. Some women, who successfully passed off an illegitimate child as their husbands’, have been said to make confessions about who the actual biological father is in the case of the child’s premature death. These unsolicited confessions are prompted by the indigenous belief that a ‘proper’ burial is one that is held within one’s own clan.

#### ii. Rights versus responsibilities

In Cornwall’s study of ‘reproductive strategies’ within one Yoruba community in Nigeria, she highlights the “socially embedded nature of reproductive agency, the contingencies that complicate ‘choice’”, and the fact that reproductive strategies are “always tempered by relations of sociality and power” in this particular setting ([19]. p252). Case studies from Igboland in Nigeria align with this assertion. Here, we specifically explore the issue of ‘social’ parenthood and how this disturbs international concepts of reproductive rights.

A woman’s (or man’s) right not to have a child until she feels ready for this responsibility seems rational enough. Without the financial, psychological, or emotional means, for instance – or even just the desire – to attend to a child, a reasonable argument is that it would be unjust to bring a child into existence in the first place. Cornwall’s study among the Yoruba, however, provides a vivid portrait of how the responsibility of raising and caring for an unplanned child is appropriated by parents and other family members who tend to see such a child as ‘theirs’ and who willingly take full responsibility for the new offspring, often being able to override the decisions/wishes of the biological parent. In this way, concepts around paternity rights or rights to abortion, for example, become redundant, having little opportunity to develop as the child is essentially taken off the biological parents’ hands. Cornwall thus emphasizes a key point about ‘the embeddedness of reproduction in the lives of a wide range of people, beyond the immediate parents of the child’ ([19]. p246).

The notion that responsibility for a child may not necessarily be seen as lying solely (or even primarily) with individual, biological parents is also demonstrated by the following personal examples. We both happen to have cases in our respective families in Igboland where the indigenous institution of ‘woman marriage’ is a contemporary reality. In one instance, an elderly woman whose grandchildren had reached adulthood was only ever able to have one child of her own. As this child happened to be female, the woman was concerned about the fact that her husband’s name ‘would not last’, or be carried on. There would be no-one to occupy their marital home as her daughter was married and living elsewhere. She thus decided to marry a wife for herself – one who would hopefully have male children to carry on the family name. She approached a cousin of one of the authors – a woman in her twenties at the time and presently in her thirties – who accepted the proposal. The bride in question had developed a chronic illness in early adulthood and was convinced that this condition would deter eligible bachelors from ever proposing to her. She therefore decided that marrying another woman was the best option for her. It would permit her to have paramours of her choice through which she would bear children. These paramours would have no claim to their biological children, however. Her children would ‘belong’ to the lineage in which she married and her ‘husband’ (i.e., the elderly woman that married her) would be the social ‘father’ of her children and bear the financial burden of caring for them.

In another instance drawn from one of our families, a marriage was contracted between a woman (currently in her early sixties, but eighteen years old at the time of her marriage) and an elderly man by his adult children. The man was a widower and in need of care which his adult children could not provide as they lived outside Igboland. The woman concerned proceeded to have children of her own. It was common knowledge that most of her children were fathered by paramours as her husband eventually grew far too old to play this role. Given the concept of social fatherhood, however, her reproductive behavior was overlooked until the number of children she had borne was considered excessive to her husband’s adult children, who also happened to have the responsibility of providing for her and her children. She was given a warning after almost ten children that any other offspring borne would not be taken as their responsibility. It was at this point that she refrained from having any more children. We were informed of a similar case in the Zambian context. One married couple, though they were relatively secure in their finances, educated, and held down professional jobs in an African capital city, depended periodically on the wife’s elder sisters for financial assistance. They had three children and were told by these sisters not to have any more, given their financial struggles. During the delivery of the fourth pregnancy, the sisters played a major role in the woman’s care, as had been their custom, and were present in the delivery room. Immediately after the delivery, they instructed the doctor to perform a tubal ligation, with their sister’s knowledge and passive consent. This was clearly a decision she was not prepared to make herself; yet in her ambivalence, she acquiesced to what she subconsciously felt made the most sense, and seemed relieved that the responsibility for making this decision was taken out of her hands. Her husband was informed after the fact, but made no protestations, either.

In these examples, there is a sense of individual rights at play (e.g., in regard to the women who had as many children as they saw fit until the financial care-takers of these children intervened), but these are clearly eclipsed by the weighting assigned to a more communal sense of rights. Although there are clearly exceptions, from our observations, this mentality does not appear to be against the norm in a range of African settings, and among a diverse range of women – whether urban- or rural-based, highly or barely educated, wealthy or poor. The mentality is particularly surprising, even to us, in the case of women that are clearly capable (financially and otherwise) of independently caring for their own children. In one way or another, there is usually a tendency, even for such women, to acquiesce to (or at least worry deeply about) certain communal conventions regarding reproduction. The concerns are rational, however, as, in addition to any personal desires to have children, one also typically desires that one’s children be secure – and a primary component of security in African contexts involves having a recognized ‘place’ in society, a concrete place of belonging, which the society itself has often pre-determined.

Vasectomies in African contexts continue to register a low rate of uptake for several reasons. One of them has to do with the assumption behind vasectomies that men’s fertility belongs to them alone, and that men’s sexuality should serve them and their wives alone. In actuality, the fertility of men in many African cultures is also regarded as belonging to the lineage. The fact that a man may have attained his personal fertility goals for his nuclear family, does not mean that his fertility is no longer ‘needed’. In several African settings, couples with infertility issues are known to turn to a close, male patrilineage member to impregnate the wife with her consent. An informal conversational interview held in 2008 with a highly-skilled, professional Nigerian (Igbo) woman in the diaspora indicates that this practice continues. Once her spouse’s infertility issues were identified, she turned to his brother to ask for his assistance in this regard. Although he was prepared to oblige to ensure that his brother had children, she later had a change of heart and eventually had a child through *in vitro* fertilization.

#### iii. ‘Things left undone’

In her article entitled *Autobiography of things left undone: Politics of literature*, *hyphenation and queered friendship in Africa*, Nyeck [20] draws our attention to the ‘things left undone’ in the analysis of African cultures: “the ‘unexpected’, the thing that is not named, not spoken ‘yet’, not fully comprehended ‘yet’, not ‘fully’ lived yet” (S. Nyeck, written communication, March 15, 2011). Same-sex sexual relationships are clearly a major thing left ‘undone’ in the African context, despite increasing attention to the issue over the last two decades. While notions of ‘culture’ can serve to severely limit the realization of sexual rights by sexual minorities, they can also serve as avenues to promote these rights, although this perspective is hardly given due consideration. Cultures have also been shown to possess a capacity to accommodate change, suggesting that there is place in most cultures for the unexpected, the things yet to be spoken about, named, or fully comprehended. Further, the community ‘safe houses’ in which individuals are indulged and guaranteed protection and a fair hearing, the socio-economic privileges that accrue to indigenes of the cultures described in this article, the notion of ‘fatherhood’ as a social, rather than purely biological phenomenon – all these are expected to be appropriated by each indigene, regardless of sexual orientation. Yet, these potentially powerful openings for the achievement of sexual rights tend to be totally overlooked and unexplored in favor of strategies that could actually serve to alienate sexual minorities from the communities that provide an important sense of belonging and identity – and that provide an uncontested bundle of rights.

Achmat alludes to this issue in his poignant analysis of the LGBTI (Lesbian, Gay, Bi-sexual, Transgender, and Intersex) movement in South Africa, where the progressive constitution (which outlaws discrimination based on sexual orientation) has naturally led to a heavy reliance on the legal system for the attainment of sexual rights:

Our courts and our laws are important and they should not be underestimated … [but] your mum is a greater part in your life than a judge – and if you [as a sexual minority] cannot convince your mum that she should love you equally, then there’s a problem. And so, even if we brought one or two parents to support our struggle, that is a greater achievement. Similarly, if we engage a traditional leader – not all of them are progressive, but one or two of them want to discuss these things – approach them and discuss with them, develop customary law and practice to become a customary law and practice that takes into account equality, diversity in our constitution. … [W]e have to know … that our parents, our family, our community [are] as much a part of us as our constitution is [21].

The tendency among some men who have sex with men in Africa, for instance, has been to align with certain facets of culture and enter into heterosexual relationships as a way of satisfying societal expectations [22]. In outlining a number of rights in this paper that pertain to all individuals, we suggest that there are other approaches to aligning with culture – approaches that can potentially ensure each individual is able to live authentically, while moving toward the full realization of rights. Kinship networks, for example, are typically not the first port of call for solutions to contemporary rights violations or barriers. Yet, we have shown that these networks can actually serve to champion specific rights and to ensure that they are realized. Referring to the various agnatic groups that constitute the most important kinship network for the Igbo indigene, Uchendu asserts:

The quality of the interpersonal relations which exist in or among these … agnatic groups differs; their respective interests are often conflicting, but from the point of view of the individual Igbo this conflict is in his own best interest. It helps him to maneuver among these agnatic groups, playing one group against the other in the interest of self-protection and social advancement ([15]. p64).

Totally ignoring certain positive cultural norms, therefore, may be tantamount to ‘throwing the baby out with the bath water’.

## Conclusions

A recent analysis of sexuality, health, and human rights, warns us against eliminating a human rights perspective that ‘defends individual bodies and their sexual pleasure and self-determination’, providing two critical reasons for this admonition: ‘First, because human beings are the most destructive species on the planet and the most likely to suffer violence and deprivation from their own kind, they need special (remedial) attention. Second, it is only from our body’s experience of pleasure and danger that we have the capacity to recognize the rights, needs, and desires of others’ ([1]. p212).

This is a compelling admonition which warrants serious consideration. Yet, when important rights are vested in the community, as in the case of the Ubang and the Igbo, the rights, needs, and desires of others can also be recognized through community experiences, and realized through engagement with communities. Building on this communal conceptualization of rights in order to realize an even wider range of rights (including sexual and reproductive health rights in particular), remains a largely unexplored strategy, however.

The brief inventory of rights examined in this paper raises at least two key issues that point to promising directions for advancing the realization of sexual and reproductive health rights in African settings. Firstly, rights, as indigenously conceived, are not necessarily contentious. This suggests that the very idea of ‘fighting for’ rights could be perceived as alien to indigenous world-views, depending on the situation. Perhaps this explains the often antagonistic reaction that activists of almost any kind are usually faced with in their bid to uphold citizens’ rights in Africa. The antagonism may have less to do with the idea of rights than with activists’ framings of this phenomenon – framings which may be divergent from local understandings of the concept. As the data in this paper demonstrate, no Igbo or Ubang indigene is granted rights or entitlements merely by virtue of his/her existence. In these indigenous contexts, human existence is not sufficient for the enjoyment or conferral of rights; rather, rights are activated (or not) in accordance with an individual’s alignment with the social structure. The rights outlined in this article are not paraphernalia which one decides to obtain or struggles to achieve. They are conferred/activated as a consequence of one’s location within social spheres. The positive slant to this notion is that the indigenous communities in question recognize that everyone has certain inalienable rights, depending on his/her location, and recognize their own role in ensuring that these rights are enjoyed. This in itself is an opening for stimulating dialogue and thinking around other possible rights and the locations in which one might most easily exercise them.

This leads to the second key issue that the findings draw our attention to – namely, the social location of rights. A purely individual approach to rights holds the danger of totally overlooking the social locations in which a range of useful rights and privileges already exists for many Africans. In not considering more seriously how to engage with social locations even as they engage with individuals, rights movements in Africa limit the parameters of their impact. Thus, our contention is not that the struggle or fight for rights is unnecessary. However, the kind of struggle that overlooks location within social institutions is troubling.

As Correa et al. assert, “Derrida is correct that human rights norms are both indispensable and insufficient; we need a human rights framework reconceived as relationally individual and social at the same time” ([1]. p211). Finding this balance between the individual and the community has been a major challenge hindering the achievement of sexual and reproductive health rights in Africa, with one receiving far more attention than the other [23]. The realization of sexual and reproductive health rights in African settings will require a myriad of strategies. Strategies that incorporate communal notions of rights as a means of attaining individual sexual and reproductive health entitlements hold promise for promoting the achievement of these rights as they are likely to resonate strongly with communities themselves, and ultimately with the individuals that often perceive themselves as being inextricably linked to the communities in question.

## Competing interests

The authors declare that they have no competing interests.

## Authors’ contributions

CU and COI conceptualized the ideas for the paper, wrote all drafts of the paper, coordinated all subsequent inputs, and contributed to the final analysis and conclusions.
